# Microvascular Dynamics: Insights and Applications

**DOI:** 10.3390/life16091537

**Published:** 2026-09-15

**Authors:** Aristotle G. Koutsiaris, Friedrich Jung

**Affiliations:** 1 MIBI Laboratory, Faculty of Medicine, School of Health Sciences, University of Thessaly, Biopolis Campus, 41500 Larissa, Greece; 2 Institute of Biotechnology, Molecular Cell Biology, Brandenburg University of Technology, 01968 Senftenberg, Germany; friedrich.jung@b-tu.de

Over three hundred years have passed since Marcello Malpighi first saw capillaries under a microscope in the 1600s. Yet reliable studies of microvascular blood flow have only recently appeared in animals and humans. A wide palette of variables affects microvascular dynamics, including vessel geometry, fluid rheology, cellular deformability, pharmacological agents, and pathological states such as sickle cell disease, microthrombosis, microvascular angina, atherosclerosis, diabetes, retinopathy, and shock. Furthermore, quantitative measures of microvascular dynamics and relevant variables, such as platelet and erythrocyte aggregation, flow velocity, and wall shear stress, act as critical clinical biomarkers to evaluate disease progression, biological functions, and treatment efficacy. Human microvascular dynamics can be affected by many conditions, but direct in vivo observations are limited to a few tissues, such as the conjunctiva (by conjunctival video capillaroscopy—CVC-), retina (by adaptive optics or optical coherence tomography angiography—OCTA-), nailfold (by nailfold video capillaroscopy—NVC-), and the sublingual tissue (by sublingual video capillaroscopy—SVC-). In this regard, in vitro, in silico, and animal modeling can shed light on cases where this is difficult in humans in vivo.

In addition, indirect methods such as laser Doppler flowmetry, laser speckle contrast imaging, and oxygen partial pressure measurements are used. For several years, microcirculation has also been assessed using contrast-enhanced ultrasound (CEUS) to visualize blood flow in the smallest blood vessels of internal organs in vivo and in real time.

This Special Issue (SI) titled “Microvascular Dynamics: Insights and Applications” closed on 18 May 2026. It was published in the journal Life (ISSN 2075-1729), belonging to the section “Physiology and Pathology”, and presented six (6) original research articles, one (1) review, and one (1) case report. It has been viewed 21,804 times (accessed on 24 August 2026). The following paragraphs present a brief overview of the articles included in the SI.

Posado-Domínguez et al. [1] focused on cancer-associated Thrombotic Microangiopathy (TMA), an underappreciated condition that typically occurs at advanced stages of cancer. In their report, a review of the pathophysiological mechanisms of cancer-associated TMA was presented, followed by five clinical cases of patients diagnosed in 2023. Various potential triggers for cancer-associated TMA were described, and a discussion regarding the necessary patient support and the different targeted therapies took place.

Many hundreds of millions of people worldwide are affected by diabetes mellitus and develop chronic microvascular impairments including nephropathy, retinopathy, and neuropathy. Liśkiewicz-Jankowska et al. [2] focused on the early detection of subclinical microvascular alterations of diabetic retinopathy by imaging techniques such as scanning laser Doppler flowmetry (SLDF), adaptive optics (AO), optical coherence tomography angiography (OCTA), and laser speckle flowgraphy (LSFG). These advanced imaging techniques do not require pupil dilation or dye injection. They also presented a very useful table and a summary of key clinical studies on retinal microvascular alterations in carbohydrate metabolic disorders after the year 2001.

Cardiovascular diseases are the leading cause of death in the developed world, and the development of atherosclerotic plaque is closely linked to them. Amun G. Hofmann [3] proposed two models for atherosclerotic plaque progression. The first model (logistic map) adopts as its primary variable the proportion of the area of an arterial lumen that is occupied by an atherosclerotic plaque and simulates its progression over time. A key parameter in this model is the growth factor *r* whοse value determines the final progression of the plaque. The second model (spatial Markov model) simulates the transition probability between states (stable or unstable plaque), incorporating variables such as inflammation levels, lipid content, shear stress, plaque burden, and the spatial dependence matrix (states of neighboring plaque regions).

Since the onset of the COVID-19 pandemic, more than 777 million confirmed cases and over 7 million deaths have been confirmed worldwide. Some survivors cannot fully recover, presenting persistent long-term symptoms that are usually described with the collective term “Long COVID”. Aristotle G. Koutsiaris [4] introduced a mathematical model for the estimation of microvascular blood supply reduction (SR) when there are quantitative data from a flat tissue area. This model takes into account both the measured microvascular hemodynamic decrease and microvascular loss, and is valid for every case–control study with relevant measurements. Microvascular loss can be estimated by vessel density reduction, foveal avascular zone enlargement, and percentage of perfused vessel reduction. The estimated SR then translates into a corresponding mass diffusion reduction of oxygen and nutrients according to the velocity-diffusion equation. For the specific case of COVID-19 (634 post-COVID patients), the estimated SR for multiple tissues and geographical areas reached a sizeable 47%. This large disruption of peripheral tissue blood supply was proposed as the principal mechanism causing Long COVID symptoms. One year later, the SR pathophysiological mechanism was directly linked to seven principal Long COVID symptoms, with a total normalized incidence of 76% [5].

Very recently, the results of two research papers confirmed the peripheral tissue hypoxia in Long COVID predicted by the SR mechanism. Silva et al. [6] reported large antioxidant enzyme depletion, increased lipid peroxidation, and reduced nitric oxide (NO) availability, with a large effect size, in a case–control study with 85 Long COVID patients. Vajdi et al. [7] reported that HIF-1α (Hypoxia-Inducible Factor 1-alpha) exhibited high network centrality in a group of 69 Long COVID patients, confirming that the hypoxia and cellular asphyxia pathways were active and closely intertwined with chronic inflammation.

Intraoperative decision-making is the real-time process in which a surgeon evaluates available information and unexpected events under the high-pressure conditions of the operating room and ultimately makes a choice. Kupke and colleagues [8] examined the performance and impact of contrast-enhanced intraoperative ultrasound (CE-IOUS) on intraoperative decision-making. CE-IOUS is a real-time imaging technique linking typical IOUS with intravenous microbubble contrast agents. The microbubble contrast agents help differentiate malignant lesions by visualizing the surrounding microcirculation. In malignant lesions, irregular hypervascularization is found in the arterial phase, as seen in hepatocellular carcinoma, or irregular marginal vascularization, as seen in metastases, most commonly colorectal metastases. Microbubbles are exhaled by the patient a few minutes after injection, with fewer side effects compared to contrast agents used for MRI or CT. Kupke et al. [8] retrospectively analyzed CE-IOUSs performed in hepatopancreatic-biliary surgery of 50 patients, and in 20 cases CE-IOUS identified an additional unexpected lesion, mostly malignant. CE-IOUS diagnostics achieved a high classification accuracy of 95.7%, substantially impacting intraoperative decision-making.

In addition, Jung et al. [9] demonstrated the use of HIFR-CEUS (High-Frame-Rate Contrast-Enhanced Ultrasound) to detect changes in the liver suspected to be malignant. With special software, an evaluation of the wash-in and washout kinetics of liver lesions is possible.

An integral part of the transplantation process is the interruption of the blood supply to the organ being transplanted. During this period of “warm” ischemia, oxygenation decreases, and the cells lose some of their functionality. Ultimately, the functional capacity of the transplanted organ will represent only a fraction of its original state. One of the strategies designed to mitigate damage during the ischemic period is ischemic preconditioning (IPC)—specifically, the controlled interruption of blood flow to the organ for short, repeated intervals before the prolonged interruption associated with transplantation. Adorjan et al. [10] simulated renal transplantation in Wistar rats by inducing total ischemia in the left kidney for 120 min, followed by a 60 min reperfusion period during which they studied metabolic, rheological, and hemodynamic parameters. Rats were divided into three groups: (1) the control (C) group, (2) the ischemia/reperfusion (I/R) group without preconditioning, and (3) the ischemia/reperfusion group with preconditioning (I/R/IPC group). Similar metabolic and hemorheological impairments were observed in both ischemic groups; RBC aggregation was significantly higher in the I/R and I/R/IPC groups in comparison to the C group. Blood flow in the renal artery and vein was significantly higher in the I/R/IPC group in comparison to the I/R group.

In hospitals, intravenous therapy with crystalloid fluids is common practice. However, colloid solutions, such as albumin, offer specific benefits and can replace or supplement crystalloids. Recent data link plasma dilution resulting from intravenous fluid administration to periodic fluctuations in plasma volume at very low frequencies (<0.001 Hz). Robert G. Hahn [11] studied plasma volume oscillations in 72 volunteers who received 3 mL/kg of hyper-oncotic albumin (20%) over 30 min. The observed plasma volume oscillations had a peak-to-peak total amplitude of 3.6–6% and a dominant frequency of 0.007 Hz (144 ± 42 min).

In microvessels, blood cannot be considered a Newtonian homogeneous fluid, and scientists are still trying to describe its flow mathematically, which has proven to be a difficult task because it depends on many parameters such as red blood cell mechanics, aggregation, local hematocrit distribution, and vascular geometry. Kaliviotis and colleagues [12] estimated apparent viscosity in vitro at various locations of the parent and daughter branches of a Y-junction with a square cross-section (50 × 50 μm). Using a recently proposed viscosity model and hematocrit profiles from earlier work for healthy and stiffened RBC suspensions, they estimated the local relative viscosity profiles η_r_(y) and the apparent viscosity (η_app_), which was considered an indicator of microvascular flow resistance.

In conclusion, we would like to thank all the authors and reviewers whose contributions helped bring this SI to completion. We would also like to acknowledge Ms. Polaris Li and Ms. Rainy Zhou from the Life Editorial Office for their valuable assistance.
